# Transfusion-Associated Hyperkalemic Cardiac Arrest in Neonatal, Infant, and Pediatric Patients

**DOI:** 10.3389/fped.2021.765306

**Published:** 2021-10-29

**Authors:** Morgan Burke, Pranava Sinha, Naomi L. C. Luban, Nikki Gillum Posnack

**Affiliations:** ^1^School of Medicine, George Washington University, Washington, DC, United States; ^2^Department of Pediatrics, School of Medicine, George Washington University, Washington, DC, United States; ^3^Division of Cardiac Surgery, Children's National Hospital, Washington, DC, United States; ^4^Children's National Heart Institute, Children's National Hospital, Washington, DC, United States; ^5^Department of Pathology, School of Medicine, George Washington University, Washington, DC, United States; ^6^Division of Hematology and Laboratory Medicine, Children's National Hospital, Washington, DC, United States; ^7^Department of Pharmacology & Physiology, School of Medicine, George Washington University, Washington, DC, United States; ^8^Sheikh Zayed Institute for Pediatric Surgical Innovation, Children's National Hospital, Washington, DC, United States

**Keywords:** transfusion, hyperkalemia, cardiac arrest, pediatric, neonate, red blood cell storage lesion, red blood cell

## Abstract

Red blood cell (RBC) transfusions are a life-saving intervention, with nearly 14 million RBC units transfused in the United States each year. However, the safety and efficacy of this procedure can be influenced by variations in the collection, processing, and administration of RBCs. Procedures or manipulations that increase potassium (K^+^) levels in stored blood products can also predispose patients to hyperkalemia and transfusion-associated hyperkalemic cardiac arrest (TAHCA). In this mini review, we aimed to provide a brief overview of blood storage, the red cell storage lesion, and variables that increase extracellular [K^+^]. We also summarize cases of TAHCA and identify potential mitigation strategies. Hyperkalemia and cardiac arrhythmias can occur in pediatric patients when RBCs are transfused quickly, delivered directly to the heart without time for electrolyte equilibration, or accumulate extracellular K^+^ due to storage time or irradiation. Advances in blood banking have improved the availability and quality of RBCs, yet, some patient populations are sensitive to transfusion-associated hyperkalemia. Future research studies should further investigate potential mitigation strategies to reduce the risk of TAHCA, which may include using fresh RBCs, reducing storage time after irradiation, transfusing at slower rates, implementing manipulations that wash or remove excess extracellular K^+^, and implementing restrictive transfusion strategies.

## Introduction

Of the nearly 14 million blood transfusions in the United States each year, over 400,000 of these were administered to pediatric patients (1, 2). A recent multicenter study found that 79% of children in the intensive care unit for cardiac conditions received at least one red blood cell (RBC) transfusion during their stay (3). Neonatal and pediatric patients are physiologically different from adults, and as such, transfusion indications and clinical management can present unique challenges (4, 5). For example, younger patients are more susceptible to electrolyte and metabolic consequences of transfusions (e.g., hyperkalemia, hypocalcemia), since the amount of blood given during surgery or massive transfusion can equal the total blood volume of a neonate, and the glomerular filtration rate of newborns does not reach maturity until near the first year of life (4, 6, 7). Moreover, many children's hospitals follow a dedicated donor strategy, wherein a single-donor unit is split into multiple pediatric packs for use in a designated patient (8–10), up to the date of expiration. This practice reduces the risk of multiple donor exposures, but with the consequence that older RBCs are then used for subsequent transfusions. Additionally, institutions that lack irradiation facilities may procure RBCs that are already irradiated off-site, which prolongs the time from irradiation to transfusion and increases the risk of transfusion-associated hyperkalemia. Accordingly, the collection, processing, and administration of RBC transfusions can influence the safety and efficacy of transfusions and contribute to adverse events (11). Indeed, a recent multicenter study found that nearly 1% of pediatric patients experience transfusion-associated hyperkalemia, which coincides with a 20% 1-day mortality rate (7) – thus, further highlighting the importance of additional study.

## The Red Blood Cell Storage Lesion

RBCs develop storage-induced damage and undergo a series of biochemical, metabolic, and structural changes that are collectively termed “*the RBC storage lesion*” [previously reviewed (12–14)]. Briefly, prolonged RBC storage results in glucose depletion and a reduction in ATP production, which impairs RBC membrane stability and increases hemolysis. Storage lesion severity can result in downstream physiological consequences that diminish both the safety and efficacy of RBC transfusions (12, 15–18). Accordingly, numerous biomarkers have been identified to monitor the pathobiological changes that red cells undergo during storage, including (but not limited to) measurements of free hemoglobin and non-transferrin bound iron, microvesicle production, 2, 3-diphosphoglyceric acid, lactate, and extracellular [K^+^] (14, 19–23). Although metabolic processes are slowed during refrigeration, hypothermic storage impairs cation transporters which alters the electrolyte composition of the RBC unit. As an example, Na^+^/K^+^ ATPase dysfunction results in K^+^ leak and accumulation within the extracellular solution of stored RBC units. Extracellular K^+^ levels increase linearly at a rate of approximately 1 mEq/L each day during refrigerated storage – until an equilibrium point is reached at ~ 60 mEq/L (24). Experimental studies by Bennett-Guerrero et al., reported a 376% increase in extracellular [K^+^] within 3-weeks of storage, exceeding the maximum level of instrument detection (20 mmol/L) (14). Clinical studies have also observed high [K^+^] in RBC units stored for longer periods of time, including a case report on the death of an infant after cardiac surgery that involved the rapid transfusion of a 32-day RBC unit with high K^+^ levels (60 mEq/L) (25, 26). The latter highlights the risk of electrolyte disturbances in transfused patients. Irradiation further exacerbates RBC K^+^ leak, as increases in oxidative stress and red cell permeability result in a two-fold increase in [K^+^] following blood product irradiation (27–29). Upon transfusion, an acute spike in serum [K^+^] can shift the myocardial resting membrane potential and trigger lethal arrhythmias (30). Indeed, transfusion-associated hyperkalemic cardiac arrest (TAHCA) is a recognized transfusion complication (31).

## Administration of RBC Transfusions

Both the RBC storage lesion and the method used to transfuse RBCs can contribute to transfusion-associated complications. RBC transfusions are administered to neonatal, infant, and pediatric patients *via* a variety of methods, including: intravenous (IV) catheter, handheld syringe infusion, or extracorporeal circulation (32–34). Conventionally, RBC transfusions are performed using a catheter, of variable bore size, placed peripherally in an accessible vein or via a central line (35). Accordingly, the route of administration can influence electrolyte homeostasis and the safety margin of this procedure. For instance, central lines are more commonly associated with hyperkalemia and cardiac arrest (25, 36). Whereas peripheral IV infusions provide ample time for transfused blood to ionically equilibrate and redistribute K^+^ with surrounding tissues before reaching the heart (35). Notably, patients with low cardiac output may still present with clinical hyperkalemia even in instances of peripheral venous administration (35, 37).

The flow rate of the transfusion can also influence the likelihood of developing transfusion-associated hyperkalemia and/or TAHCA. Transfusions with rapid flow rates can increase red cell hemolysis (32, 38) and also elevate K^+^ levels quickly with inadequate time for equilibration. Miller et al., observed that hemolysis increases with handheld syringes, resulting in a serum K^+^ concentration that exceeds the threshold for arrhythmogenicity (32). While there are guidelines for the maximum transfusion rate in pediatrics, in clinical practice, the rate of infusion is largely determined by the rate of blood loss and indication for transfusion. In the operating room or emergency setting, the use of handheld syringes is a common practice for neonatal and infant transfusions (32, 38), but can introduce considerable variability in the speed of administration compared to automated pumps. Emergency circumstances that require fast rates of transfusion also decrease the allowable time for K^+^ redistribution, which further increases the risk of TAHCA (37).

Transfusion-associated complications can also occur in pediatric populations following large volume RBC transfusions. Pediatric patients with congenital cardiac malformations often undergo reconstructive surgery that requires the use of extracorporeal membrane oxygenation (ECMO). Although adult patients can usually accommodate the volume of blood required to prime an ECMO circuit, this volume can exceed the total blood volume of a neonatal or infant patient. Consequently, pediatric ECMO patients receive large volumes of RBCs (42–105 ml/kg/day), which increases the risk of circulatory overload and hyperkalemia (34, 39). Parshuram et al., reported a case in which a 3-week old ECMO patient experienced TAHCA within a few min of infusing RBC's irradiated 10 days prior as part of the circuit prime; a blood gas measurement denoted 9 mmol/L [K^+^] at the time of cardiac arrest (40). Hyperkalemia has also been observed in cardiopulmonary bypass (CPB) procedures, especially when irradiated blood products are used in smaller patients (<5kg) (41, 42). Indeed, a single RBC transfusion can double an infant's plasma [K^+^] (26, 43). Further, both ECMO and CPB circuits place additional mechanical stress on circulating RBCs from the tubing, connectors, cannulas, and/or roller pump. This mechanical stress can alter the membrane properties of RBCs, by increasing red cell fragility and hemolysis (44–47). A study by the Naval Blood Research Laboratory found that 15% of red cells were damaged within 24-h of extracorporeal circulation (47). Saline washing of RBC's is yet another form of mechanical stress that has been shown to increase hemolysis and osmotic fragility (48). Accordingly, fresh RBCs are preferred for neonatal and infant patients undergoing cardiac surgery, ECMO, or an exchange transfusion (49–51).

## Consequences of Transfusion-Associated Hyperkalemia

**Figure 1:**
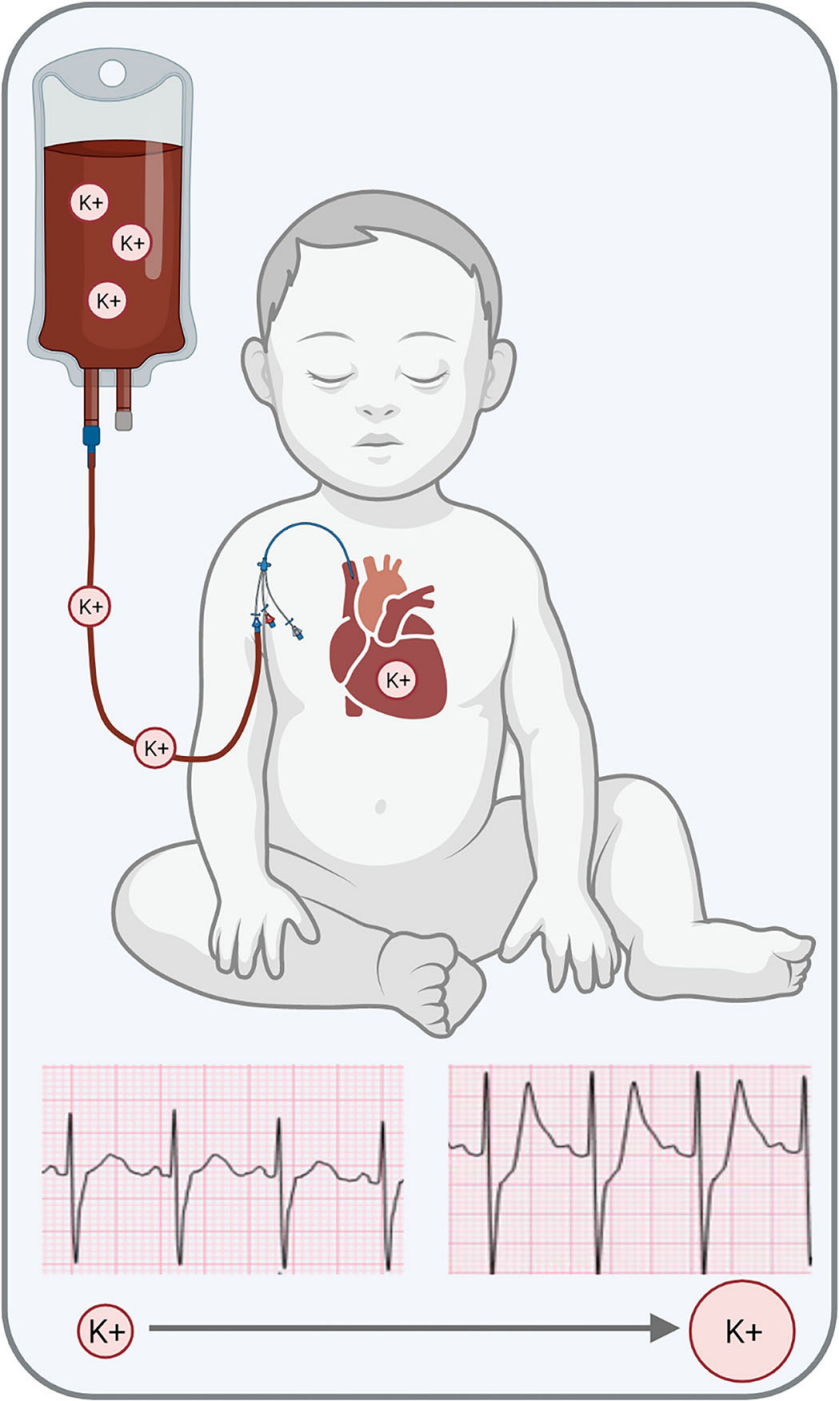
Transfusion-associated hyperkalemia can precipitate cardiac electrical disturbances, which can present with peaked *T*-waves in the electrocardiogram. Further increase in serum [K+] can result in cardiac arrest. Graphic generated in BioRender. ECG traces kindly provided by Dr. Elizabeth Sherwin.

Blood transfusions are life-saving procedures; nevertheless, RBC transfusions are associated with a wide range of complications. The latter includes increased postoperative complications and infections, impaired postoperative recovery, longer hospital stay, and increased morbidity following cardiac surgical procedures (52–60). As previously discussed, RBC transfusions can also precipitate hyperkalemia (26), as the extracellular solution of stored RBC units can reach 40–70 mM [K^+^] (19, 25). Indeed, more than 75% of critically ill (adult) patients and 18–23% of trauma (pediatric) patients experience elevated serum K^+^ following RBC transfusion (61, 62). Upon transfusion, high K^+^ levels can shift the myocardial resting membrane potential and precipitate adverse electrophysiological outcomes, from a short-lasting atrial flutter to protracted ventricular fibrillation (30, 63). An acute increase in serum [K^+^] can delay electrical conduction in the atrioventricular node, His bundle, and Purkinje system, which manifests as a prolonged PR interval and widened QRS complex (64). Hyperkalemia also predisposes the heart to pathological conditions, including ventricular tachycardia, ventricular fibrillation, atrioventricular block, and asystole (30, 63, 64). In some cases, cardiac electrical instabilities may be detectable in electrocardiogram recordings (65, 66) before the onset of severe events (Figure 1), such as cardiac arrest that require immediate intervention (25, 67). As highlighted above, risk factors for TAHCA may include the volume and rate of RBC transfusion, storage age of blood products, and irradiation of red cells – although the perceived risk of TAHCA remains debated (68).

Recently, our laboratory conducted the first experimental study to show a direct correlation between red cell storage duration, hyperkalemia, and cardiac electrophysiology (69). Using intact heart preparations and human induced pluripotent stem cell-derived cardiomyocytes, we reported that RBC storage age was associated with increased extracellular [K^+^], reduced cardiac automaticity, and slowed electrical conduction. Notably, cardiac electrophysiology parameters remained stable following exposure to fresh RBC units, but older products precipitated bradycardia, impaired sinus node function, and delayed atrioventricular conduction. The latter suggests that the storage age of blood products may be a modifiable risk factor for hyperkalemic cardiac arrest and warrants further investigation.

Although TAHCA is an adverse event that is anticipated by anesthesiologists, it can still prove difficult to manage. In a simulation study (70), only one-fourth of anesthesia residents suspected hyperkalemia as an underlying cause of pulseless electrical activity, and only one-third of residents correctly resuscitated patients using the correct pediatric dose of epinephrine. Further, a separate case series found that serum [K^+^] during or immediately after cardiac arrest ranged from 5.9–9.2 mEq/L and that TAHCA had poor in-hospital survival of only 12.5% (71). To improve patient outcomes, it is important to examine and understand how the composition of transfused blood product(s), the method of transfusion administration, and patient demographics (31, 72) can increase susceptibility to TAHCA. Unfortunately, transfusion recipients often have several comorbidities, which may result in underreporting of transfusion-associated adverse events (73).

## TAHCA in Neonatal, Infant, and Pediatric Patients

The prevalence of transfusion-associated hyperkalemia and/or TAHCA is currently unknown, as reports are limited to case studies and case reports. Nevertheless, hyperkalemia from blood transfusion is the second most common cause of perioperative cardiac arrest in neonates, infants, and children as documented by the perioperative cardiac arrest registry, second only to hypovolemia from blood loss (74). In Table 1, we have summarized 24 case studies and reports of TAHCA in neonatal, infant, and pediatric patients – with data reported as indicated in the original article (25, 37, 39, 40, 66, 67, 71, 75–92). We also highlight a case report in which TAHCA was avoided by pre-transfusion K^+^ filtration (87). Briefly, our literature search identified TAHCA case reports and case series using the following search query: “hyperkalemia or hyperkalemic” AND “transfusion” AND “cardiac arrest” AND “neonate OR neonatal OR infant OR pediatric” in both PubMed and Google Scholar databases. Only reports on patients ≤18 years old were included. Studies were excluded if transfusion-associated hyperkalemia was not explicitly associated with cardiac arrest, or if cardiac arrest was attributed to another condition. A manual search of the cited references was also performed.

**Table 1:** Pediatric cases of transfusion-associated hyperkalemia cardiac arrest.

**Patient age, genetic sex, weight, procedure**	**Transfusion volume, method of delivery**	**Blood storage age & irradiation**	**[K+] in unit (mmol/L)**	**[K+] in patient before and after transfusion (mmol/L)**	**Patient response**	**Patient outcome**	**References**
2-month-old, female, weight not reported. Cardiac surgery.	The patient received 120 mL of RBCs. Central venous line.	Blood storage age: 6 days Irradiation: 48 h prior to infusion	55.3	Before: 4.1 After: 6.3	Patient went into cardiac arrest 10 min into transfusion. Patient received CPR and cardiac defibrillation.	Survived	Baz et al., (67) (Lebanon)
72-h-old, male, ~3.5 kg. Exchange transfusion.	The patient received 500 mL of RBCs. Umbilical vein.	Blood storage age: <21 days old Irradiation: not reported	9.55	Before: not reported After: 5.16	Patient went into cardiac arrest during the transfusion. Resuscitation measures were not reported.	Survived	Bolande et al., (75) (United States)
<72-h-old, male, ~3.5 kg. Exchange transfusion.	The patient received 485 mL of RBCs. Umbilical vein.	Blood storage age: <21 days old Irradiation: not reported	22.7	Before: 5.4 After: 5.3	Patient went into cardiac arrest during the transfusion. Resuscitation measures were not reported.	Survived	
<72-h-old, female, ~3.5 kg. Exchange transfusion.	The patient received 500 mL of RBCs. Umbilical vein.	Blood storage age: <21 days old Irradiation: not reported	21.1	Before: 3.95 After: 6.55	Patient went into cardiac arrest shortly after the transfusion. Resuscitation measures were not reported.	Deceased	
<72-h-old, genetic sex not reported, ~3.5 kg. Exchange transfusion.	The patient received 320 mL of RBCs. Rapid injection via umbilical vein.	Blood storage age: <21 days old Irradiation: not reported	20.5	Before: 3.75 After: 7.75	Patient went into cardiac arrest shortly after the transfusion. Resuscitation measures were not reported.	Deceased	
2-day-old, genetic sex not reported, 2.26kg. Venoveno ECMO.	1 RBC unit to prime the circuit. Right internal jugular vein central venous catheter.	Blood storage age: 5 days Irradiation: yes	28.1	Patient before: not reported After: 9.7	Patient went into cardiac arrest at the start of ECMO. Patient received external cardiac massage.	Survived	Bolton et al., (76) (United Kingdom)
9-year-old, male, weight not reported. Pulmonary laceration after trauma.	Transfusion of 1L of RBCs rapidly followed by 500 mL of RBCs slowly. Method of delivery was not reported.	Blood storage age: units were 13, 13, and 14 days old respectively Irradiation: not reported	10.2, 7.5, and 13.0, respectively	Before: not reported After: not reported	Patient received 500 mL of 10% glucose in water with 15 units of regular insulin.	Deceased	Bostic and Duvernoy (77) (United States)
6-month-old, genetic sex not reported, 7 kg. Unspecified surgery.	Volume not reported. The patient received rapid administration of reconstituted whole blood via large bore intravenous access.	Blood storage: 13 days old Irradiation: not reported	Not reported	Before: 4.2 After: 9.6	Patient developed ventricular tachycardia intraoperatively during RBC transfusion. Patient received epinephrine, bicarbonate and CPR.	Outcome not reported	Brown et. al., (37) (Canada)
1.5-year-old, genetic sex not reported, 7 kg. Unspecified surgery.	Volume not reported. The patient received rapid administration of reconstituted whole blood via large bore intravenous access.	Blood storage: 13 days old Irradiation: not reported	Not reported	Before: 5.0 After: 7.3	Patient went into cardiac arrest intraoperatively during RBC transfusion. Patient received CPR.	Outcome not reported	
1-year-old, genetic sex not reported, 8 kg. Unspecified surgery.	Volume not reported. The patient received rapid administration of reconstituted whole blood *via* large bore intravenous access.	Blood storage: 13 days old Irradiation: not reported	Not reported	Before: 3.1 After: 7.5	Patient developed ventricular tachycardia intraoperatively during RBC transfusion. Patient received epinephrine and CPR.	Outcome not reported	
2-year-old, genetic sex not reported, 11 kg. Unspecified surgery.	Volume not reported. The patient received rapid administration of reconstituted whole blood *via* large bore intravenous access.	Blood storage: 21 days old Irradiation: not reported	Not reported	Before: 4.3 After: 12.3	Patient developed asystole intraoperatively during RBC transfusion. Patient received CPR.	Outcome not reported	
2-year-old, genetic sex not reported, 12 kg. Unspecified surgery.	Volume not reported. The patient received rapid administration of reconstituted whole blood via large bore intravenous access.	Blood storage: >17 days old Irradiation: not reported	Not reported	Before: 5.0 After: 9.0	Patient went into cardiac arrest intraoperatively during RBC transfusion. Patient received epinephrine and CPR.	Outcome not reported	
17-year-old, genetic sex not reported, 33 kg. Unspecified surgery.	Volume not reported. The patient received rapid administration of reconstituted whole blood via large bore intravenous access.	Blood storage: >18 days old Irradiation: not reported	Not reported	Before: 5.3 After: 7.3	Patient went into cardiac arrest intraoperatively during RBC transfusion. Patient received CPR.	Outcome not reported	
13-year-old, genetic sex not reported, 70 kg. Unspecified surgery.	Volume not reported. The patient received a rapid transfusion of RBC concentrate diluted in 250mL of normal saline.	Blood storage: 7 days old Irradiation: not reported	Not reported	Before: 3.4 After: 6.4	Patient went into cardiac arrest intraoperatively during RBC transfusion. Patient received CPR.	Outcome not reported	
12-month-old, male, 7.85 kg. Craniosynostosis repair.	1.5 units of RBCs over 2 h, then 2 units of RBCs over 45 min, finally 20 mL of RBCs. Peripheral venous line, final 20mL was delivered via handheld syringe.	Blood storage age: 3, 17, 21, and 22 days old, respectively Irradiation: The first unit was not irradiated, the last three units were irradiated 14 days prior to transfusion	8.2, 30.1, >40, and >40, respectively	Before: 3.1 After: 10.1	Patient went into cardiac arrest 15 min after 20 mL of RBC via syringe was administered. The patient received external chest compressions, adrenaline 1mg, atropine 0.4mg, calcium chloride 10% 25ml, Calcium Gluconate 10% 5ml, and Sodium Bicarb 8.4% 20 ml.	Survived	Buntain and Pabari (78) (Australia)
7-year-old, female, 20 kg. Hip surgery.	15 units of RBC over 4 h, then 100 mL of whole blood over 10 min. The method of delivery was not reported.	Blood storage age: Whole blood was 16 days old. Age RBC units not reported. Irradiation: not reported.	Not reported	Before: 3.7 After: 10.3	Patient went into cardiac arrest 10 min after 100 ml of whole blood was transfused. Patient received external Cardiac massage CaCl2, NaHCO3, and regular insulin.	Deceased	Chen et al., (79) (Taiwan)
Fetus, genetic sex not reported, weight not reported. Intracardiac transfusion.	Volume not reported. Transfusion directly into the left ventricle of the heart.	Blood storage age: four-day-old gravity sedimented packed cells Irradiation: not reported	24.7 mmol/L	Not reported	Not reported	Not reported	Galligan, et al., (80) (Saudi Arabia)
2-week-old, male, 3.2 kg. Cardiac surgery.	2 units of RBC < 5 days old during CPB, 2 units of RBC < 5 days old over 3 h post op, then 60 mL RBC 32 days old over 10 min. Central venous catheter.	Blood storage age: 2 units of RBC < 5 days old during CPB, 2 units of RBC < 5 days old, 60 mL RBC 32 days old. Irradiation: not reported	32-day old unit ~ 60	Before: 4.2 30 min before transfusion of 32-day-old blood: 4.0 During cardiac arrest: 8.9 mmol/L After: not reported	Patient went into cardiac arrest immediately after the second transfusion of 32-day old blood. Patient received a cardiac massage.	Deceased	Hall et al., (25) (United States)
7-month-old, female, weight not reported. Ventricular septal defect repair.	3 units of RBCs in mannitol-adenine-phosphate. Extracorporeal circuit	Blood storage age: stored 8–10 days post irradiation Irradiation: yes	Not reported	Before: not reported After: 10.6 mEq per L.	CPR	Survived	Inaba, et al., (81) (Japan)
1-day-old, female, 1.4 kg. Left pneumonectomy.	Two doses (10mL each) of fresh umbilical cord blood. Handheld syringe. Central venous catheter.	Blood storage age: fresh Irradiation: not reported	8.0	Before: not reported After: 5.0	Cardiac arrest shortly after transfusion. Patient treated with 0.5mL of 8.5% calcium gluconate.	Deceased	Inoue et al., (82) (Japan)
4-month-old, female, 6.65 kg. Surgical correction for anomalous origin of left coronary artery.	320 mL of RBCs. Cardiopulmonary bypass circuit.	Blood storage: not reported Irradiation: not reported	Not reported	Before: 4.3 After: 10.2	Patient went into cardiac arrest after the surgery while still on CPB. Resuscitation measures included sodium bicarbonate, glucose, insulin, and calcium chloride.	Survived	Ivens et. al., (83) (Belgium)
2 h old, male, 3.23 kg. Anemia.	35 mL of RBCs. Method of delivery was not reported.	Blood storage age: not reported Irradiation: not reported	Not reported	Before: >9.0 After: 9.8	Patient went into cardiac arrest and an exchange transfusion was initiated.	Survived	Kavčič et al., (84) (Slovenia)
5-month-old, male, 3.6 kg. Surgical repair of H-type tracheoesophageal fistula.	60 mL of RBCs over 2.5 h. Arterial line.	Blood storage age: 3-4 days Irradiated 60 min prior	Not reported	Before: 3.8 After: 4.9	Cardiac arrest 1 h after start of transfusion. Patient was treated with furosemide.	Survived	Kang et al., (66) (United States)
9-month-old, female, 0.7 kg. ECMO.	Extracorporeal volume of an ECMO circuit (~260 ml). Catheter inserted directly into right atrium via pulmonary artery (16Fr), and (12F) venous drainage cannula.	Blood storage age: not recorded 3-days post gamma irradiation	35	Before: not reported After: 9.0	Cardiac arrest immediately after starting ECMO. CPR performed, calcium gluconate treatment.	Survived TAHCA, but later died of condition	Kim, et al., (39) (Korea)
11-year-old, female, 49 kg. Mitral valve replacement.	1 unit of RBCs. Cardiopulmonary bypass circuit.	Blood storage age: 9 days old Irradiation: not reported	>20.0	During cardiac arrest: 9.9 After cardiac arrest: 4.9	Patient went into cardiac arrest shortly after the transfusion. Resuscitation measures included Furosemide and sodium bicarbonate.	Survived	Martin et al., (85) (Peru)
3 patients, <18-years-old, weight not reported. Unspecified surgery.	Volume reported as “massive transfusion” Method of delivery not reported.	Blood storage age: not reported Irradiation: not reported	Not reported	Not reported	Not reported	(1) Survived; (2) Deceased	Morray, et al., (86) (United States)
10-month-old, female, 7.3 kg. Liver transplant.	665mL of RBCs. Method of delivery was not reported.	Blood storage age: not reported Irradiation: yes	Before filtering: 16.9 After filtering: 1.9	Before: not reported After: not reported	Patient did not go into cardiac arrest because of potassium filter.	Survived.	Nakagawa et al., (87) (Japan)
3-week-old, male, 8 kg. Venovenous hemofiltration.	146 mL of blood primed CVVH. Central venous catheter.	Blood storage age: 21 days old Irradiation: 10 days prior to transfusion	14.3	Before: 4.0 After: 9.0	Patient went into cardiac arrest during initiation of blood primed CVVH. Patient received 3 boluses of 0.1 mg/kg Epinephrine, 100% O2, 200 mg/kg CaCl2, and 3 mmol/kg NaHCO3.	Survived	Parshuram and Cox (40) (Canada)
Neonate, male, weight not reported. Exchange transfusion.	350 mL of RBCs. Peripheral venous access.	Blood storage age: <24 h old Irradiation: not reported	Not reported	Before: not reported After: 4.5	Patient went into cardiac arrest shortly after RBC administration. Patient received a cardiac massage.	Survived	Pew (88) (United States)
28-h-old, male, 1.1 kg. Exchange transfusion for disseminated intravascular coagulation.	160 mL of RBCs. Method of delivery not reported.	Blood storage age: <48 h old Irradiation: not reported	13.0	Before: 4.6 After: 12.0	Patient went into cardiac arrest during the conclusion of the exchange transfusion.	Deceased	Scanlon and Krakaur (89) (United States)
14-year-old, male, weight not reported Resection of spinal cord tumor.	3 units of RBCs	Blood storage age: not reported Irradiation: not reported	Not reported	Before: 4.6 After: 7.9	Patient developed pulseless electrical activity. Patient received chest compressions and epinephrine.	Survived	Smith, et al., (71) (United States)
9-year-old, female, weight not reported. Anterior spine instrumentation for severe scoliosis.	5 units of RBCs	Blood storage age: not reported Irradiation: not reported	Not reported	Before: 4.1 After: 7.9	Patient developed pulseless electrical activity. Patient received chest compressions, atropine, and epinephrine.	Survived	
12-year-old, male, weight not reported. Anterior spine instrumentation for severe scoliosis.	6 units of RBCs Method of delivery was not reported.	Blood storage age: not reported Irradiation: yes	Not reported	Before: 3.9 After: 7.1	Patient developed asystole. Patient received chest compressions and epinephrine.	Deceased	
2-day-old, male, 2.7 kg. Craniotomy for Arnold Chiari III in premature infant.	350 mL of RBC. Method of delivery was not reported.	Blood storage age: not reported Irradiation: yes	Not reported	Before: 3.6 After: 5.9	Patient developed asystole. Patient received chest compressions and epinephrine.	Deceased	
17-year-old, male, weight not reported. Trauma.	39 units of RBCs Method of delivery was not reported.	Blood storage age: not reported Irradiation: not reported	Not reported.	Before: 3.1 After: >8	Patient developed asystole. Patient received chest compressions, epinephrine, bicarbonate, and calcium.	Deceased	
1-month-old, male, 3.1 kg. Surgical repair of hemorrhagic mass in left posterior fossa.	28 units of RBCs. Double lumen (4Fr) venous catheter in the right subclavian vein.	Blood storage age: not recorded Irradiation: 5-days post	19.9	Before: 3.7 After: 14.2	Cardiac arrest shortly after rapid injection of 80 ml RBCs. Patient treated with 10 U insulin, 50 ml of 50% dextrose, multiple doses of epinephrine, 0.1 mg atropine, 30 mg CaCl2 and chest compressions.	Deceased	Sohn et al., (90) (Korea)
1-h-old, female, 2.22 kg. Exchange transfusion.	384 mL of RBCs during the first exchange, then 6 h later received another 290 mL of RBCs. That 22 h received yet another 70 mL of 7-day old blood. Peripheral venous access.	Blood storage age: Third unit was 7 days old, the other units age was not reported. Irradiation: not reported	>20.0	Before: 4.3 After: 12	The first cardiac arrest happened a few moments after the second volume exchange. The second cardiac arrest occurred 32 minutes after the third exchange. Patient received 2 mL of Calcium glucoheptonate each time.	Deceased	Taylor et al., (91) (Canada)
1-h-old, male, 2.16 kg. Exchange transfusion.	185 mL of RBCs in 15 mL aliquots. Peripheral venous access.	Blood storage age: 5 days old Irradiation: not reported	Third exchange unit: 9.7	Before: 6.9 After: 7.6	Patient went into cardiac arrest immediately after the injection of the aliquots. Patient received 1.5 mL calcium gluconate.	Survived	
16-year-old, male, 29.6 kg. Anteroposterior spine fusion.	9 units of RBC over 90 min. Peripheral venous access.	Blood storage age: not reported Irradiation: not reported	Not reported	Before: not reported After: 12.0	Patient went into cardiac arrest 9 h into the surgery. Patient received chest compressions.	Survived	Woodforth (92) (Australia)

Within the identified TAHCA case reports, patient's age ranged from 1 h to 18 years old; with 24 patients <1 year old, and 16 reports of TAHCA in neonatal patients. Roughly half of these studies included transfusions that were carried out during surgical procedures such as cardiac surgery, trauma, spinal surgery, hip surgery, organ transplant, and tracheoesophageal fistula repair. The remaining studies included medical transfusions such as ECMO and exchange transfusion. Fifteen reports included transfusions that were initiated centrally through a venous catheter and thirteen reports included transfusion *via* a peripheral vein. As observed in Table 1, there was a non-uniform method of reporting lab values between case studies and reports. In the case of Morray et.al., the authors reported that there were three cases of TAHCA, but did not provide any additional details beyond two of the three patients suffering fatal arrhythmias (86).

A number of clinical case reports have documented TAHCA in patients receiving large volume transfusions, although we have also highlighted reports of TAHCA following rapid, small volume transfusions of older and/or irradiated blood products (66, 67, 82, 84, 88, 89, 91). Inoue et al., observed peaked T-waves immediately after administering 10 mL of blood via handheld syringe to a 1.4kg newborn (82). In this specific case, blood was administered *via* a central venous catheter, which can facilitate the delivery of a higher K^+^ load to the heart without allowing adequate time for redistribution. Similarly, Taylor et al., reported that an infant developed hyperkalemia and cardiac arrhythmia after administering 5-day old blood in small 10 mL aliquots (91). One possible explanation for developing clinical hyperkalemia after small volume injections is the higher rate of hemolysis when smaller needles are used and/or fast rates of transfusion (32, 93). Other cases included transfused patients who received fresh blood without incident, but later developed TAHCA after the administration of older and/or irradiated RBCs that were stored for >24 h (25, 78, 79, 91). As an example, Hall et al. reported the successful transfusion of 4 units of fresh blood to a 2-week-old male undergoing cardiac surgery without incident; upon transfusion of 60 mL of 32-day old blood delivered via a central line, the patient suddenly developed cardiac arrest and died (25). In this case, the older blood products were administered due to a shortage of fresh units. Similarly, Buntain et al., reported successful transfusion of 1.5 units of RBCs in a craniofacial surgery, but, later the patient developed cardiac arrest following the administration of 60 mL of 22-day old blood that had been irradiated 14-days prior (78). Kim et al., reported a case of a 9-month old patient with severe hypoxia receiving ECMO support without incident for several days (39). In this case, the ECMO circuit was primed with RBCs <24 h post-irradiation. However, after an oxygenator failed, a new ECMO circuit was primed with donor blood that had been stored 3-days post-irradiation. Immediately after restarting ECMO, the patient went into cardiac arrest. ECMO is the equivalent of a massive transfusion for pediatric patients, who are vulnerable to large fluctuations in electrolyte concentrations; moreover, in this clinical case a catheter was inserted directly into the right atrium of the patient, which minimized the diffusion potential of the K^+^ load. In each of the described clinical cases, prolonged blood storage time (with or without irradiation) may have contributed to the development of TAHCA, as the extracellular [K^+^] within the RBC unit increases linearly with time and doubles 24-h post-irradiation (27).

We also noted clinical practices to avoid TAHCA using pre-transfusion manipulations. Nakagawa et al., reported the avoidance of a hyperkalemic event by using a K^+^ adsorption filter (87). In this case, a 10-month old patient was undergoing a liver transplant that required a massive transfusion, which was initiated with an in-line filter to reduce the [K^+^] from 16.3 to 1.9 mEq/L. Additionally, Sohn et al., demonstrated the applicability of a continuous autotransfusion system (CATS) to reduce the K^+^ level in donor blood before transfusion (90). In this case, the patient received 1 unit of non-irradiated blood, followed by another unit of blood that was 5-days post-irradiation. The patient went into cardiac arrest shortly after the transfusion. After restoring normal cardiac function, the surgical team utilized CATS to prevent TAHCA during the transfusion of another 26 RBC units. These strategies may be useful in situations where fresh RBC units are in short supply.

## Potential Mitigation Strategies to Reduce the Incidence of Hyperkalemia

Strategies to reduce the incidence of TAHCA include the use of fresh blood products, reducing storage time post-irradiation, slower rates of transfusion, and other manipulation techniques. Current standard of practice dictates that blood units <5 days old and within 24 h of irradiation are employed in massive transfusion (50, 94); although older RBC units are administered if fresh units are not readily available. Fresh RBC units are also preferred for neonatal and pediatric cardiac surgery (95). Fresh whole blood (<48 h) has been used for cardiac surgery at a few select institutions (96), although whole blood is not routinely available to most transfusion services. For children receiving ECMO, consensus panels recommend the use of fresh RBCs within 5-days of collection and irradiated RBCs are used within 24-h (50). This recommendation stems from the lack of evidence-based data required to reach consensus on the safety of older RBCs in the context of critically ill children (34, 97). Finally, blood sparing and blood conservation procedures are recommended to reduce the number and volume of RBCs transfused to neonatal, infant, and pediatric patients (98).

RBC washing with a non-plasma solution is another potential mitigation strategy, which reduces the extracellular [K^+^] to a physiologically-compatible level (99, 100). This strategy has been adopted in cardiac surgery cases requiring cardiopulmonary bypass. Swindell et al., reported that pre-washing irradiated RBCs reduced [K^+^] from >20 to 0.8 mmol/L (42). Further, 36% of patients receiving unwashed, irradiated RBCs had a serum [K^+^] >6 mmol/L during cardiopulmonary bypass compared to 0% of patients receiving pre-washed RBCs. Pre-bypass ultrafiltration can also normalize electrolyte levels and reduce the risk of hyperkalemia. Delaney et al., reported that ultrafiltration significantly reduced [K^+^] from 10.9 to 6.0 mEq/L in ECMO circuits primed with fresh RBCs (101). Utilization of K^+^ adsorption filters to normalize electrolyte levels during extracorporeal support is another potential mitigation strategy, as in-line filters can remove >90% of extracellular K+ while maintaining flow rates up to 50 mL/min (81, 87, 102).

## Conclusion

Transfusion-associated hyperkalemia is a recognized complication of massive and rapid RBC transfusions in neonates, infants, and children (103). Based on available case reports, clinical hyperkalemia can occur when aged and/or irradiated blood products are transfused, and when blood products are administered *via* a central line vs. a peripheral site (25, 36, 71). Further, the use of handheld syringes with small bore needles can increase hemolysis at the injection site and is a potential cause of transfusion-associated hyperkalemia in emergency situations (32, 38). Although there is debate around whether transfusion-associated hyperkalemia causes cardiac arrest and increases the incidence of TAHCA, there is no doubt that an increased serum [K^+^] can exacerbate a precarious physiological state in transfused patients (26, 83). While the prevalence of TAHCA is not known and may be underreported (73); we have highlighted 24 case reports and case series that identify potentially fatal outcomes if appropriate precautions are not taken to remedy electrolyte imbalance. TAHCA is not unique to neonatal and pediatric populations (61, 71), but these patients have increased susceptibility due to their unique physiology. There is a 20% 1-day mortality rate in pediatric patients who experience TAHCA (7), and hyperkalemia after transfusion is the second most common cause of perioperative cardiac arrest in neonates, infants, and children (74). Potential strategies to mitigate the risk of TAHCA include the use of fresh RBCs for pediatric cardiac surgery and ECMO, limiting the duration of storage post-irradiation, washing RBCs and/or pre-bypass filtration to remove extracellular K^+^, slower rates of transfusion, and the use of specialized pre-transfusion filters to reduce [K^+^] (42, 81, 87, 93, 101, 102, 104). To abrogate TAHCA in the future, additional studies are warranted to evaluate the safety and efficacy of these mitigation strategies and/or implementation of restrictive transfusion strategies (97, 105). Indeed, restrictive transfusion strategies can also help to mitigate blood supply shortages that have resulted from the COVID-19 pandemic, due in part to fewer blood donations (106).

## Author Contributions

MB and NP performed a literature search and generated Table 1. MB, NP, NL, and PS drafted and approved the manuscript. All authors contributed to the article and approved the submitted version.

## Funding

This work was supported by the National Institutes of Health (R01HL139472 to NP), Children's National Heart Institute, Sheikh Zayed Institute for Pediatric Surgical Innovation, and the Children's National Research Institute.

## Conflict of Interest

The authors declare that the research was conducted in the absence of any commercial or financial relationships that could be construed as a potential conflict of interest.

## Publisher's Note

All claims expressed in this article are solely those of the authors and do not necessarily represent those of their affiliated organizations, or those of the publisher, the editors and the reviewers. Any product that may be evaluated in this article, or claim that may be made by its manufacturer, is not guaranteed or endorsed by the publisher.
